# Large Carbon Dioxide Fluxes from Headwater Boreal and Sub-Boreal Streams

**DOI:** 10.1371/journal.pone.0101756

**Published:** 2014-07-24

**Authors:** Jason J. Venkiteswaran, Sherry L. Schiff, Marcus B. Wallin

**Affiliations:** 1 Department of Earth and Environmental Sciences, University of Waterloo, Waterloo, Ontario, Canada; 2 Department of Geography and Environmental Studies, Wilfrid Laurier University, Waterloo, Ontario, Canada; 3 Department of Ecology and Genetics/Limnology, Uppsala University, Uppsala, Sweden; DOE Pacific Northwest National Laboratory, United States of America

## Abstract

Half of the world's forest is in boreal and sub-boreal ecozones, containing large carbon stores and fluxes. Carbon lost from headwater streams in these forests is underestimated. We apply a simple stable carbon isotope idea for quantifying the CO_2_ loss from these small streams; it is based only on in-stream samples and integrates over a significant distance upstream. We demonstrate that conventional methods of determining CO_2_ loss from streams necessarily underestimate the CO_2_ loss with results from two catchments. Dissolved carbon export from headwater catchments is similar to CO_2_ loss from stream surfaces. Most of the CO_2_ originating in high CO_2_ groundwaters has been lost before typical in-stream sampling occurs. In the Harp Lake catchment in Canada, headwater streams account for 10% of catchment net CO_2_ uptake. In the Krycklan catchment in Sweden, this more than doubles the CO_2_ loss from the catchment. Thus, even when corrected for aquatic CO_2_ loss measured by conventional methods, boreal and sub-boreal forest carbon budgets currently overestimate carbon sequestration on the landscape.

## Introduction

Boreal and sub-boreal ecozones are large (about 17 million km^2^) and about half the world's forest cover [1]. Forests in these zones contain large carbon stores and contribute significant fluxes in the global carbon budget [2]. Part of the carbon fixed from the atmosphere by forests is returned to the atmosphere via aquatic surfaces [3]–[6]. In the past, this flux has been ignored in the construction of carbon budgets for forested watersheds and is now predicted to be higher than expected [7]. In Sweden, annual estimates of carbon sequestration in forests are 10% lower if aquatic carbon losses are included [8]; similar amounts are expected for Norway [9]. In the USA, stream and river CO_2_ loss may be five times greater than previously thought [10]. The difficulty in accounting for the aquatic loss lies in inadequate information on the enormous number of small headwater streams with high CO_2_ concentrations. In boreal Sweden, 90% of total stream length is in catchments less than 15 km^2^ [11].

Neglecting the aquatic export of terrestrially fixed carbon (via dissolved inorganic carbon (DIC), CH_4_, particulate organic carbon (POC), and dissolved organic carbon (DOC)) in landscape carbon budgets results in overestimating net ecosystem exchange by 25% to 70% [12]–[14]. The active pipe model for inland waters [15] increased estimates of total terrestrial export by two-fold while concluding that CO_2_ loss to the atmosphere was necessarily underestimated because small streams were entirely excluded for lack of emission and distribution data [3]–[7], [10]. Subsequently, ‘in-stream heterotrophy' was added to the stream portion of the budget [5] but other DIC sources, e.g., DIC-rich groundwaters, are poorly known and therefore excluded. The knowledge gap between the significant contribution of headwater streams in a basin and the small amount of data about them has been termed *aqua incognita* [11].

All streams in an extensively studied 4th order Swedish boreal catchment were supersaturated in CO_2_ [16]. There, CO_2_ loss from stream surfaces directly to the atmosphere was 30%–50% of the dissolved carbon (DIC+DOC) exported from the forest (2.9 gC/m^2^/yr of 9.8 gC/m^2^/yr) [17], [18]. In an Alaskan headwater stream network, greatest variability and mean fluxes of CO_2_ were in the first order streams [19] with 9.0 gC/m^2^/yr lost from the entire Yukon River basin [20].

In sub-boreal catchments in Canada [21] and a peat catchment in northeast Scotland [13], CO_2_ loss from stream surfaces was 36% and 34% of the dissolved carbon exported from the catchments. These studies noted that gas loss from stream surfaces to the atmosphere is significant, but all neglected the higher CO_2_ evasion rates from stream surfaces upstream of their sampling locations. Without good estimates of CO_2_ fluxes, ecosystem-scale metabolism calculations also contain this uncertainty and bias. DIC in headwater streams is a net result of a number of processes, including: dissolution of carbonate and weathering of some minerals in soils and bedrock, in-stream biotic respiration and fixation, exchange with atmospheric CO_2_, and shallow groundwater input [17], [22]–[25]. Although it is commonly assumed that CO_2_ saturation is controlled by mineral weathering and in-stream biotic activity in carbonate systems, boreal forests are, in many cases, characterized by high-DIC shallow groundwaters derived from terrestrially respired organic carbon [8], [24].

Here, we focus on silicate bedrock catchments because they constitute 45% of boreal catchments [1] and we avoid the complications of the contribution of significant amounts of DIC from carbonate [26]. Silicate-dominated boreal landscapes contribute significantly to global carbon fluxes [27]. Headwater streams in these catchments have low to moderate pH values and moderate to high concentrations of CO_2_ that is modern in radiocarbon terms [28], [29]. Thus, DIC from shallow groundwater is directly related to soil and root respiration instead of long-term mineral weathering.

In undisturbed, shaded, nutrient-poor, silicate-bedrock, boreal headwater streams, shallow groundwater is the major source of DIC since in-stream respiration is low relative to gas exchange with the atmosphere [30]–[33]. Furthermore, in silicate terrain, shallow groundwater retains the 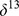
C value of the DIC input from DOC and soil OC decomposition and exhibits modern radiocarbon ages [28], [29] indicating weathering is unimportant at this scale. Thus for small streams of up to a few hundred metres length in this landscape, soil and root respiration is the key 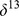
C-DIC source.

Degassing of CO_2_ from these streams causes the 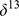
C-DIC to increase due to well known equilibrium isotopic fractionation between CO_2_, HCO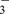, and CO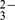 and during CO_2_ loss to the atmosphere — since the 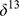
C- CO_2_ value is less than the bulk 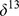
C-DIC value, the loss of CO_2_ will cause the remaining 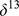
C-DIC value to increase [34], [35]. Additionally, since CO_2_ loss does not affect carbonate alkalinity, there is a concomitant increase in pH as degassing occurs along the stream [36]. This CO_2_ loss also includes CO_2_ lost as groundwater transits the riparian zone adjacent to the stream since these DIC and 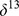
C-DIC changes are indistinguishable from those in the stream proper. Recent work presented a moderately complex modelled formulation of the degassing effects on 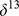
C-DIC and focused on several French streams [35].

At a much broader level, we can use the characteristic degassing trajectories of decreasing DIC vs increasing 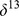
C-DIC to estimate (1) how far along the degassing curve a particular sampling site in a stream is and (2) how much CO_2_ loss there must have been upstream of the sampling site. This gives us the opportunity to use discrete samples from a stream to estimate the upstream CO_2_ loss without direct knowledge of the DIC concentration of the groundwater end-member. Since groundwater 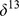
C-DIC can be easily constrained based on C3 plants and soil organic matter formation [22], [28], [37] in silicate-dominated catchments, the average groundwater DIC concentration can then be determined as a result of the modelling process. Discrete samples are available from the mouths of streams where whole-lake mass balances have been completed [38], along stream networks [16], , and potentially from archived samples and data sets [40]. Because only in-stream samples are needed, this technique makes it possible to cover large catchment areas. Logistically, headwater stream CO_2_ fluxes can be rapidly assessed without the large investment required to study only one catchment in detail.

Given the (1) poor knowledge of the extent of headwater stream surface area (lengths and widths [11], [24], but see recent methods using digital elevation models [18], [20] and branching theory [41]) and (2) narrow constraint on 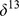
C-DIC in groundwater, we suggest these small streams can be studied with archived and new samples. We place the results in a context of landscape-scale degassing trajectories where 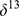
C-DIC of groundwater, stream, and small lakes all differ. We focus on improving CO_2_ loss estimates from headwater stream surfaces in two catchments: (1) Harp Lake, Canada where stream CO_2_ loss is situated in a catchment C budget and (2) Krycklan, Sweden where stream CO_2_ losses are higher than previously estimated.

## Materials and Methods

### Site description

Two field sites of contrasting vegetation and location were selected: Harp Lake catchment, Canada (45°22′ N, 79°08′ W) and Krycklan catchment, Sweden (64°14′ N, 19°50′ E). Three headwater streams in the Harp Lake catchment were studied. All are underlain by the gneissic bedrock of the Canadian Shield [42] with mixed forests of sugar maple (*Acer saccharum*), beech (*Fagus grandifolia*), yellow birch (*Betula alleghaniensis*), white pine (*Pinus strobus*), and aspen (*Populous tremuloides*). Harp Lake catchment receives 1033 mm/yr precipitation. Mean annual air temperature is 5°C. Stream catchments ranged from 3.7 km^2^ to 21.7 km^2^ with stream lengths from 170 m to 760 m. All streams were first or second order and catchments had varying amount of wetland cover (<0.3% to 8.5%) [43], [44].

Stream samples from around Harp Lake were collected from July 1989 to November 1991 across various flow conditions near the mouth of each stream (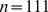). DIC samples were collected in Pyrex culture tubes with polycone caps and analysed colorimetrically as per standard methods in the Ontario Ministry of the Environment lab at Dorset, Ontario, Canada. Samples for 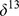
C-DIC analysis were collected in 500 mL glass bottles with polycone caps and analysed by standard off-line dual-inlet IRMS techniques. Groundwater was sampled from piezometers near the middle of the Harp 4–21 catchment (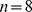) [28], [29]. An automated titrator (PC-Titrate Man-Tech Associates) was used to measure pH. Stream temperature was measured in the field. Stream DOC concentrations varied with stream flow with a median concentration of 5.1 mgC/L (range of 1.1 mgC/L and 17.2 mgC/L) and flow-weighted annual average around 10 mgC/L [45], [46]. Harp Lake epilimnion was sampled from March 1990 to November 1991 for DIC concentration and 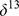
C-DIC (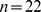).

This work was initially conducted to study the origin, transport, cycling, and fate of organic carbon from soils to DOC and DIC in a soft water catchment by combining 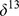
C and 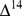
C analyses [28], [29], [42], [43], [45], [46]. DOC turnover rates in streams, lakes, and wetlands are fast, <40 yr, and on the same time scale as acid deposition in the area. There is extensive DOC cycling in upper soil layers and the source of DOC to headwater streams changes seasonally with water table level. Soil CO_2_ and 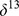
C- CO_2_ profiles show characteristic patterns of root respiration, decomposition of SOM and diffusional gradients along with modern radiocarbon ages.

The Krycklan catchment is underlain by metagreywacke bedrock and has forests of Norway spruce (*Picea abies*) and Scots pine (*Pinus sylvestris*), but deciduous trees are commonly found in the riparian zone of larger streams. It receives 600 mm/yr precipitation. Mean annual air temperature is 1°C. Stream catchment areas ranged from 0.03 km^2^ to 67 km^2^ and stream lengths from 0.02 km to 96.5 km. Streams were first to fourth order and had varying amounts of wetland cover (0% to 40%) [16].

Stream samples were collected in June, August, and November 2006 (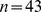). All samples were collected in septum-capped, screw-topped glass vials and analysed via headspace equilibration by gas chromatography or on-line GC-IRMS. Shallow groundwater was sampled from suction lysimeters along a transect parallel to the lateral flow paths towards one of the headwater streams (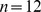). Stream temperature was measured in the field. Typical annual pH range of was 3.7 to 6.3 in headwaters and 5.7 to 7.4 in 4th order streams. First-order-stream DOC concentrations were 5.0 mgC/L to 40.0 mgC/L and in 4th order streams were 5.0 mgC/L to 15.0 mgC/L [47].

Research at Krycklan catchment is focused on integrating water quality, hydrology, and ecology in flowing waters [48]. Some recent work includes characterizing the loss of CO_2_ from stream surfaces by stream size and season [16], [47]. Streams were always supersaturated in CO_2_, greatest in the headwaters, and negatively correlated with pH [16]. Owing to the importance of the gas exchange coefficient in controlling CO_2_ loss rates and its requirement for scaling across landscapes, it was independently measured across streams in Krycklan catchment [47]. The source of the excess CO_2_ was largely explained as respired carbon being exported from catchment soils [49]. As part of this work, 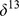
C-DIC values can be used to identify CO_2_ degassing, compared with the labour-intensive flux measurements, scaled flux estimates, and to asses the upstream CO_2_ loss that is demonstrated to be large and important [18].

### Calculations

We built a simple, parsimonious degassing model in Matlab (MathWorks, Natick MA USA) for high-DIC, low-pH, silicate headwater streams. Only DIC concentration, pH, 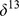
C-DIC and temperature are required to be measured in the stream since all other carbonate species concentrations and 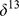
C values can be calculated. The model partitioned total DIC into CO_2_, HCO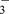, and CO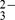 according to pH and temperature-dependent acid dissociation constants and equilibrium [50], [51] and kinetic [52] fraction factors as summarized by [34]. The calculations here are similar to those employed in CO2sys [53], seacarb [54], and streamCO_2_-DEGAS [35] but do not require as many input parameters, many of which are not available for our catchments. In small headwater streams, this simple approach may be appropriate to survey a large area. Larger, more detailed models may be required for larger and longer streams and rivers, but it becomes more difficult to measure all variables required to ground-truth such models.

Key assumptions to this approach are that streams are nutrient-poor, low pH, with low community metabolic rates relative to stream velocity and gas exchange, and catchments have C3 vegetation, silicate bedrock, with high DIC shallow groundwater reflective of the vegetation and soils. Both Harp Lake catchment [21], [28], [29], [42]–[46], [55] and Krycklan catchment [16], [24], [47], [56]–[58] meet these assumptions. Both have low nutrient concentrations, pH values, and associated metabolic rates under undisturbed forest canopies. Stream lengths and segments are on the order of hundreds of metres and water travel times are less than an hour. Forests in these catchments contain only C3 vegetation, are sub-boreal and boreal, and are underlain by crystalline silicate bedrock. Groundwaters are acidic and DIC-rich, as outlined below. This approach is generalizable to the plethora of small streams that meet these criteria across the silicate bedrock areas of the sub-boreal and boreal forests such as the headwaters of catchments on the Canadian and Fennoscandian/Baltic Shields. Source code is provided so that additional components can be added to address the complexities of carbon cycling as needed if the basic assumptions for field sites are not met, such as larger streams with longer travel times, large diel temperature variability, the confluence of several stream sections, measured rates of in-stream metabolism, known patterns of groundwater discharge, or the influence of carbonate-rich bedrock following portions of the streamCO_2_-DEGAS model [35], which has been used in larger rivers. Further, model sensitivity to variability in input parameters may be easily assessed with Monte Carlo approaches.

The chemical equilibrium calculations can be easily recreated in any modelling language. Since ionic strength of these soft waters is very low (conductivity is typically 15–40 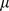
S), the activity coefficients were assumed to be approaching unity. This time-forward model differs from [35] in that: (a) carbonate dissolution was not included since our catchments are on silicate bedrock; (b) total alkalinity calculations differ slightly; (c) DIC concentration is the basis for calculations rather than total alkalinity; (d) the model starts with a small plug of shallow groundwater discharged to the surface; (e) groundwater is continuously added to the modelled stream if there are corresponding flow measurements; and (f) the iterative process to find a best fit between measured stream values and modelled results.

Though there is some contribution of organic acids to total alkalinity [59], loss or gain of CO_2_ does not change organic or carbonate alkalinity. Here we model alkalinity based on the carbonate system since in high-DIC, low-pH waters the main cause of pH change is CO_2_ loss and assume that the contribution of organic alkalinity to pH is relatively constant and minor.

In practice, to calculate the fraction of CO_2_ lost by the sampling point in the stream, DIC concentration, pH, and 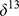
C-DIC measurements in the stream are required. An inverse modelling approach is used as the time-forward model takes initial constrained estimates of groundwater DIC, pH, and known 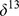
C-DIC, and allows CO_2_ to degas with time. The resulting values of stream DIC, pH, 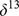
C-DIC, and 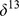
C- CO_2_ with time are outputs. The model was iteratively re-run with Matlab's *fminsearch* function to reduce the sum of squared errors between measured stream DIC, pH, and 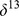
C-DIC and modelled values. The resulting best-fits then provide the average groundwater DIC concentration and the amount of CO_2_ that was lost upstream of the sampling point. In this way, an average groundwater DIC concentration can be determined. Additionally, the groundwater 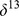
C-DIC must be determined but this value is highly constrained by plant and soil 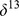
C values, as above, and can be confirmed with a number of piezometers [28], [29]. Matlab code to run the model forward and inversely is available from the corresponding author and https://github.com/jjvenky/CO2-from-headwater-streams.

At each time step, a small portion of CO_2_ was removed from the modelled aquatic system via gas exchange:
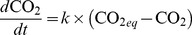
(1)


where 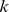 is the gas exchange coefficient, 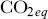 is the CO_2_ equilibrium concentration determined with the Henry's constant [50], and CO_2_ is the solvated CO_2_ concentration. This necessarily reduced the DIC concentration since:
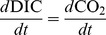
(2)

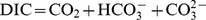
(3)


The remaining DIC was then re-apportioned to its constituent species by determining the pH (as 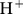) required to hold the carbonate-based alkalinity constant [36], [60]:
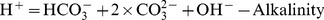
(4)


The 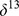
C portion of the model employed well-known temperature-dependent isotopic fractionation factors between DIC species, and solvated CO_2_ and atmospheric CO_2_ [34]: at 4°C, these are kinetic gas exchange fractionation (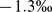) since the 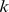 for ^13^CO_2_ is slightly slower than for ^12^CO_2_, equilibrium fractionation (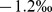) between atmospheric CO_2_ and solvated CO_2_, CO_2_–HCO_3_^−^ fractionation (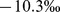), and HCO_3_^−^–CO_3_^2−^ fractionation (+3.3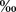). At each time step, the 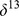
C value of each species was calculated. Typical values of input parameters and calculated parameters are summarized in Table 1.

**Table 1:** Typical model input and calculated parameters.

Parameter	Typical Value	Unit	Notes	Citation
*Typical model input parameters (* *Fig. 2* * and raw data)*				
DIC	1200	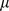 mol/L	Typical measured value	
pH	5.5		Typical measured value	
Temperature	4	 C	Typical measured value	
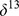 C-DIC	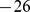	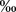	Typical measured value	
*Calculated parameters based on model inputs above*				
Alk	103	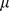 eq/L	Carbonate alkalinity held constant	
CO_2_	1098	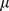 mol/L	Calculated from DIC, pH, log 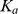 , log 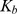	
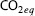	25	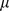 mol/L	Calculated from log 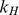 and 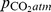	
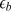	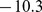	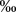	CO_2_*–HCO_3_^−^, function of temperature	[34], [ 50]
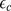	3.3	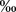	HCO_3_^−^–CO_3_^2−^, function of temperature	[34], [ 52]
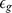	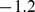	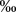	CO_2_– CO_2_, function of temperature	[34], [ 50]
log 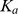	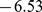		CO_2_–HCO_3_^−^, function of temperature	[68]
log 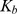	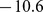		HCO_3_^−^–CO_3_^2−^, function of temperature	[69]
log 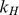	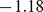	log(mol/L/atm)	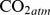 – CO_2_, function of temperature	[50]
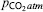	380	ppmv	Partial pressure of CO_2_ in the atmosphere	
p 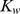	14.8		Function of temperature	[70], [ 71]

The effects of continuous groundwater input to the modelled stream that significantly changes the stream volume over the reach of interest can also be included by adding an amount of water with known or estimated initial groundwater values (DIC, 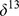
C-DIC, pH, alkalinity) at each time step. The amount can be determined by stream length and discharge measured at the catchment outlet or as the difference in flow between two sites. This comprised an additional input parameter used during best-fit modelling of the H4-21 data since this is a small first-order stream, the stream length is known, and the discharge was measured at the stream mouth along with DIC, 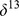
C-DIC, and pH. Here, groundwater input was assumed to be constant down the reach. In each case, the CO_2_ lost was multiplied by the measured daily discharge and reported relative to the catchment area.

### Permissions

Headwater streams in the Harp Lake catchment were studied under the auspices of pre-existing Ontario Ministry of the Environment research program. The Krycklan catchment is a part of the Svartberget LTER site run by the Swedish University of Agricultural Sciences and the area is developed for scientific purposes. Specific permissions for these activities were not required. No endangered or protected species were involved in either site.

## Results and Discussion

**Figure 1:**
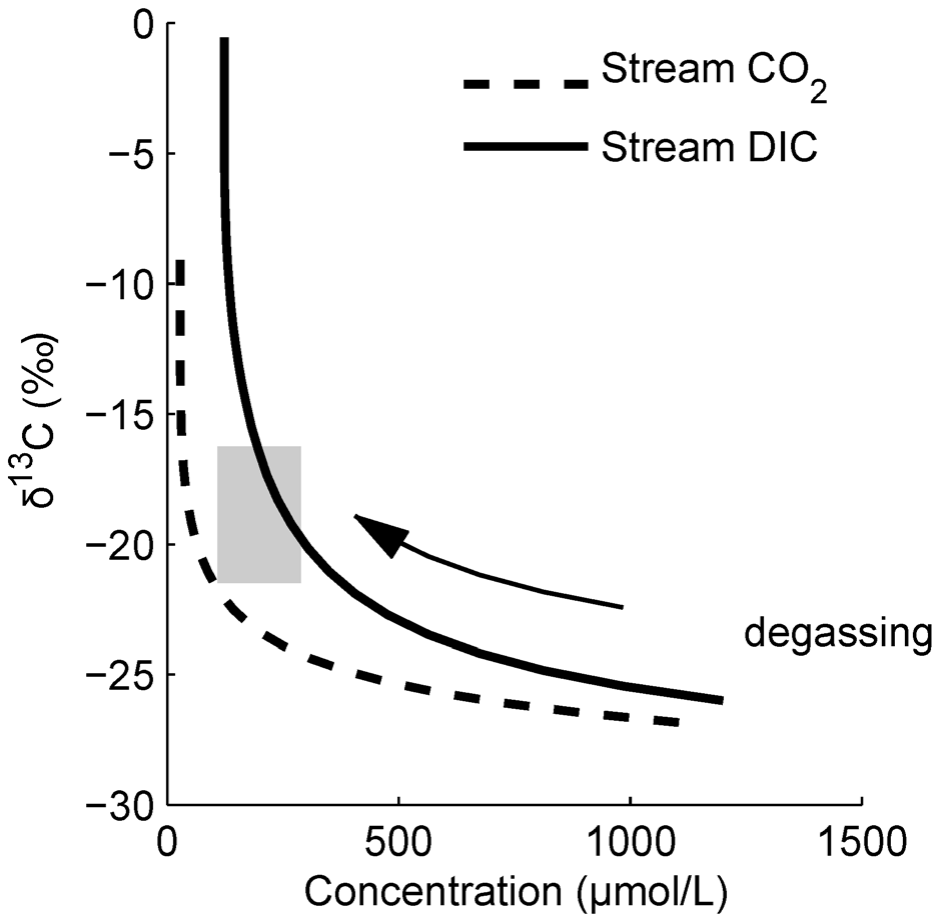
DIC and CO_2_ degassing trajectories show that as CO_2_ is lost from streams, 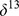
C values increase at different rates for DIC and CO_2_. 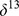
C-DIC is typically measured, not 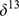
C- CO_2_, but the 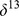
C of both DIC and CO_2_ during CO_2_ loss can be modelled using well known isotope fractionation factors. For this example, a typical groundwater end-member was chosen with initial DIC of 1200 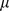
mol/L, 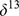
C-DIC of 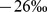, pH of 5.5, and thus carbonate alkalinity of 103 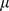
C- CO_2_, but the 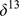
C of both DIC and CO_2_ during CO_2_ loss can be modelled using well known isotope fractionation factors. For this example, a typical groundwater end-member was chosen with initial DIC of 1200 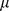
mol/L, 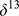
C-DIC of 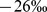, pH of 5.5, and thus carbonate alkalinity of 103 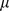
eq/L. In watersheds, variation in the groundwater end-member results in a ‘family’ of curves with similar trajectories. The grey box represents the 25th and 75th percentiles of DIC and 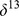
C-DIC collected from three different first- and second-order streams draining into Harp Lake, Ontario, Canada. Samples were collected at the mouth of each stream from July 1989 to November 1991 (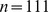). Significant CO_2_ loss (60% to 80%) must have occurred by the sampling point in each stream in order for the box to fall where it does on the degassing curve.

As CO_2_ is initially lost from a stream, there is little associated change in 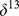
C-DIC (Fig. 1). Only after about half of the DIC has been lost is there an observable change of about 2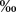 in 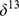
C-DIC. In streams where measured 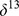
C-DIC values are significantly different than shallow groundwater, a large amount of CO_2_ must have already been lost from the stream by the time the sample was collected (Fig. 1). Thus, CO_2_ flux measurements obtained with data from an individual sampling site where the 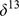
C-DIC value is several per mille greater than the groundwater 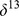
C-DIC value must underestimate stream and catchment CO_2_ loss rates since the stream surface with higher CO_2_ concentrations and higher flux rates is upstream of such a sampling site. The degree of underestimation needs to be better quantified because these results suggest that net ecosystem exchange and C storage may be lower than assumed in boreal and sub-boreal ecozones.

**Figure 2:**
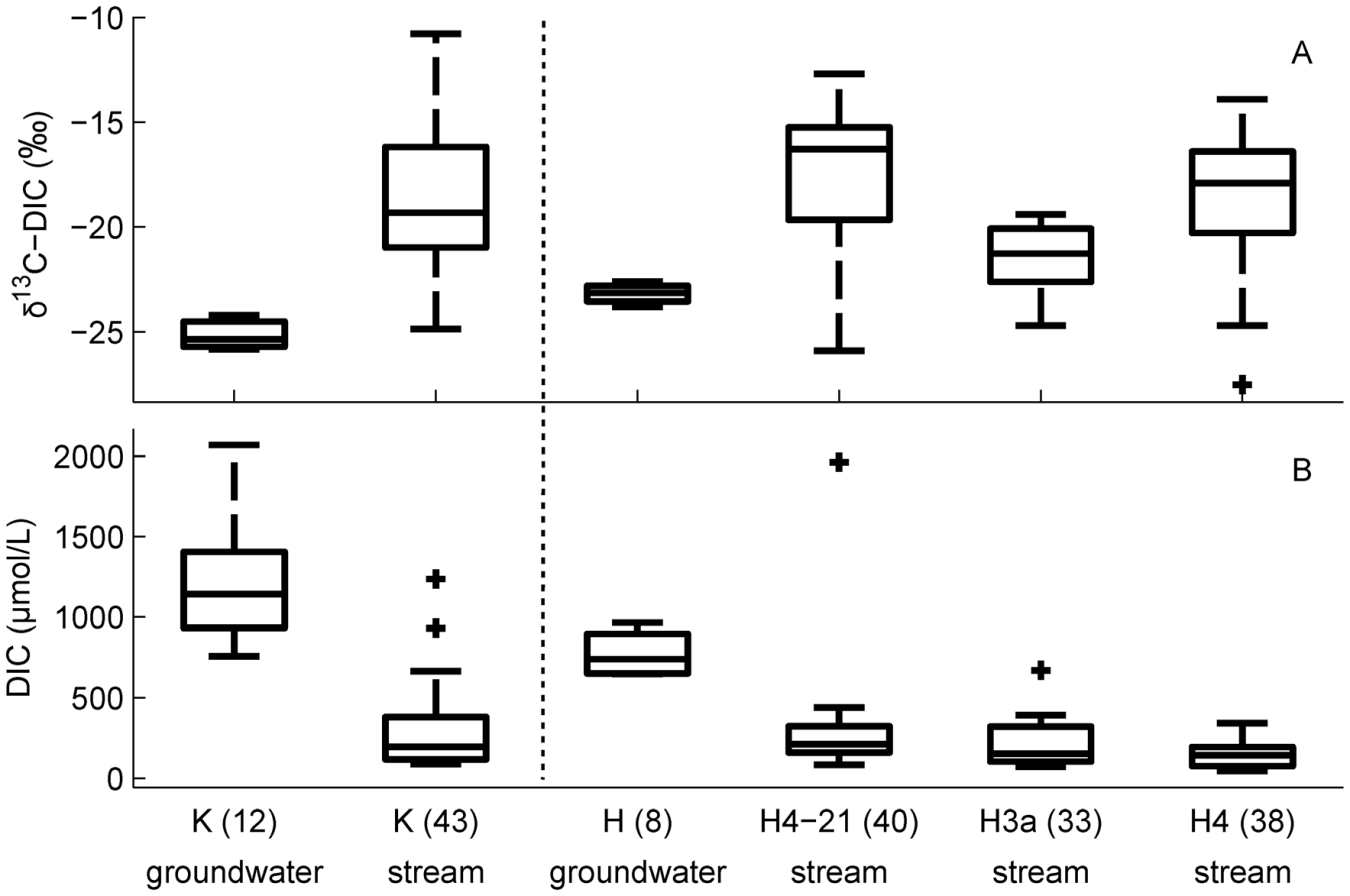
Groundwater and stream (a) 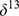
C-DIC values and (b) DIC concentrations from headwater catchments in Sweden (left) and Canada (right). Groundwater and stream data are from the headwater stream network in Krycklan catchment, Sweden (K) and three first- or second-order streams draining into Harp Lake, Ontario, Canada (H with stream numbers; [43], [44]). The boxes represent 25th and 75th percentiles, mid-line the median, whiskers the most extreme datum not outside 1.5
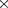
inter-quartile range (IRQ), and + the data outside 1.5
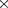
IQR. Number of samples is indicated in parentheses. At these two sites where we have both groundwater and stream data, there is a clear increase in 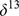
C-DIC values from groundwater to stream sampling points along with a large decrease in DIC concentration.

We applied these ideas to a suite of headwater stream data from Krycklan catchment in Sweden and Harp Lake in Canada for which both groundwater and stream measurements were available. Stream DIC concentrations were lower and 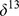
C-DIC and pH values greater than their respective groundwater values in both catchments (Fig. 2). The range of 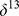
C-DIC in shallow groundwater, 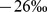 to 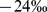 is tightly constrained by the narrow range of 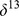
C in C3 plants and soil. This is comparable to measured 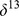
C-DOC values [28] so in-stream respiration would produce DIC with the same 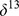
C-DIC value as shallow groundwater. Surface water was allowed to lose CO_2_ via gas exchange and the chemical and isotopic equilibria were adjusted accordingly (see Methods).

There are many groundwater discharge patterns possible. In small streams, the difference in discharge measured at two locations may be the only way to estimate groundwater inputs. As such, it may be difficult to parameterize [43]. In a stream network in Alaska, high resolution CO_2_ and discharge data suggest variable and varying groundwater inputs [19] along the lengths of the streams. The net effect of groundwater input to small streams is to increase stream DIC concentration, reduce the 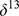
C-DIC value, decrease pH, and make the stream appear closer to the initial shallow groundwater values (Figs 1 and 2).

**Figure 3:**
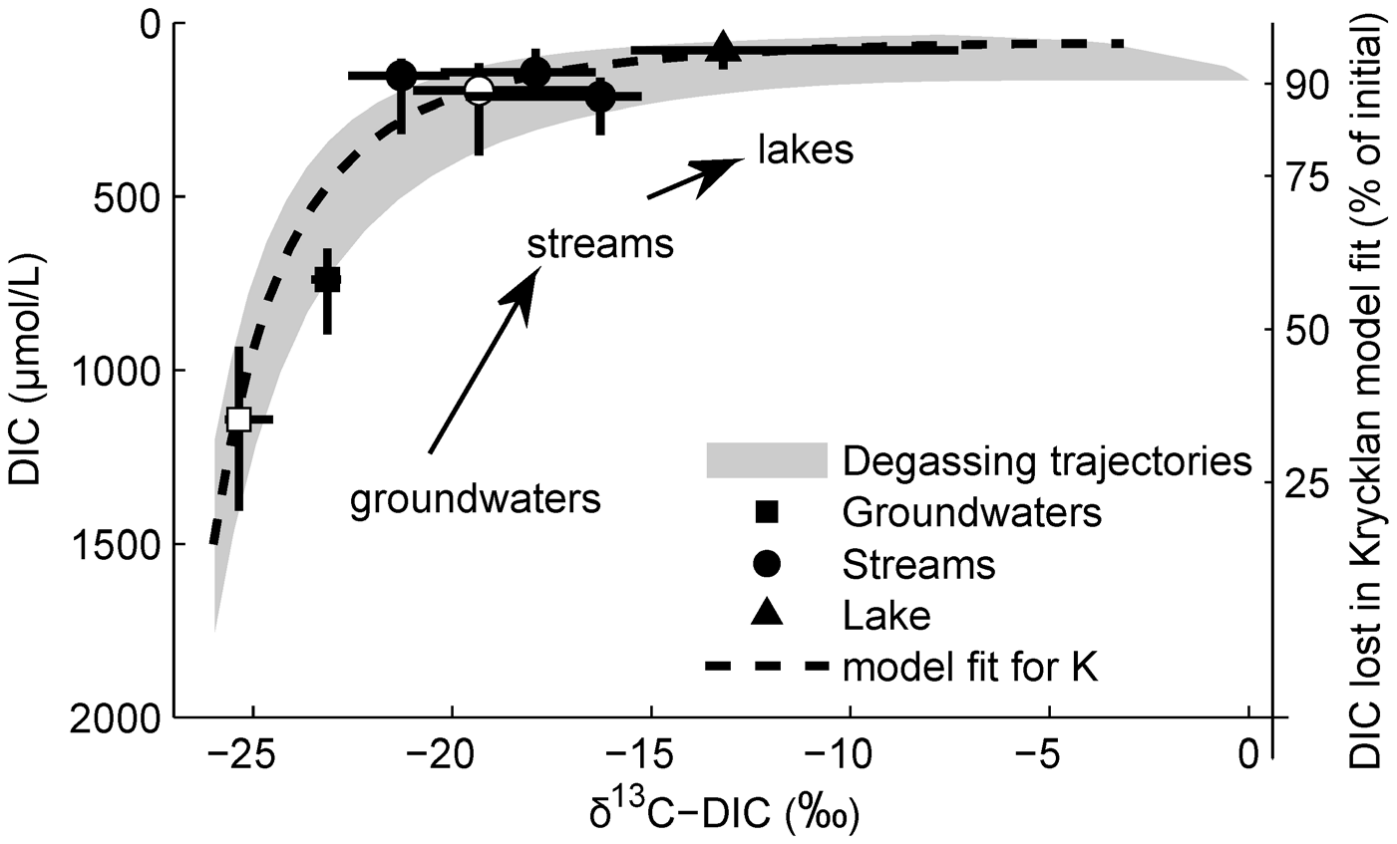
Typical DIC and CO_2_ degassing trajectories (grey band) show that small initial increases in 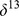
C-DIC values signify large losses in CO_2_ from groundwater to streams. The 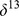
C progress along this trajectory is a natural result of CO_2_ degassing, not other processes (e.g., primary production, mineral weathering, etc.). The grey band represents the combined trajectories of degassing curves with a combination of initial DIC concentrations (1200 to 1800 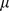
mol/L), 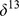
C-DIC (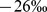) and pH values (4.0 to 5.5). The grey band also describes the landscape-scale trajectory of DIC concentrations and 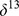
C-DIC values from shallow groundwater discharging into headwater streams and degassing while flowing downhill to small lakes. Groundwater, stream, and lake data from the two study sites are displayed as 25th and 75th percentiles (lines) with medians (symbols). Harp Lake data are from the epilimnion from March 1990 to November 1991 (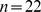). The line represents the degassing trajectory that was fit to the median of the Krycklan (open symbols) dataset. The secondary *y*-axis indicates the amount of DIC lost along the degassing model fit for the Krycklan site.

While shallow groundwaters in boreal forest catchments vary, to some extent in DIC concentration, 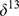
C-DIC, pH, and temperature, the effect of changing these variables does not alter the fundamental relationship between 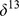
C-DIC and DIC during CO_2_ degassing. Increased initial DIC concentration, lower pH, and lower alkalinity cause the sigmoid-like relationship to approach its plateau 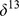
C-DIC value more quickly and thus requires a greater loss of CO_2_ before 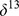
C-DIC values will increase appreciably. This is demonstrated in the range of landscape-scale degassing trajectories (grey area in Fig. 3). This range is akin to a sensitivity analysis by confirming the shape of the DIC vs 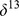
C-DIC relationship across a wide range of DIC concentrations and pH values. Buffered groundwaters discharging into headwater streams exhibit a shallower curve than acidic, poorly buffered waters. The degassing trajectories in DIC vs 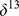
C-DIC space describe the CO_2_ loss from groundwaters to streams and ultimately to headwater lakes as decreasing DIC, increasing 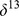
C-DIC, and increasing pH (Fig. 3).

Scaling-up fluxes estimated via these methods to catchment- and landscape-scale requires areas or lengths of headwater streams, catchment areas, or groundwater discharge areas. Here, we avoid using stream length and surface area, parameters not easily obtained in headwater catchments, but see [18], [20], and instead report stream losses relative to their catchment area. In this manner, the CO_2_ loss from aquatic surfaces is easily compared in the same units used to assess net ecosystem exchange and productivity at the catchment scale.

**Figure 4:**
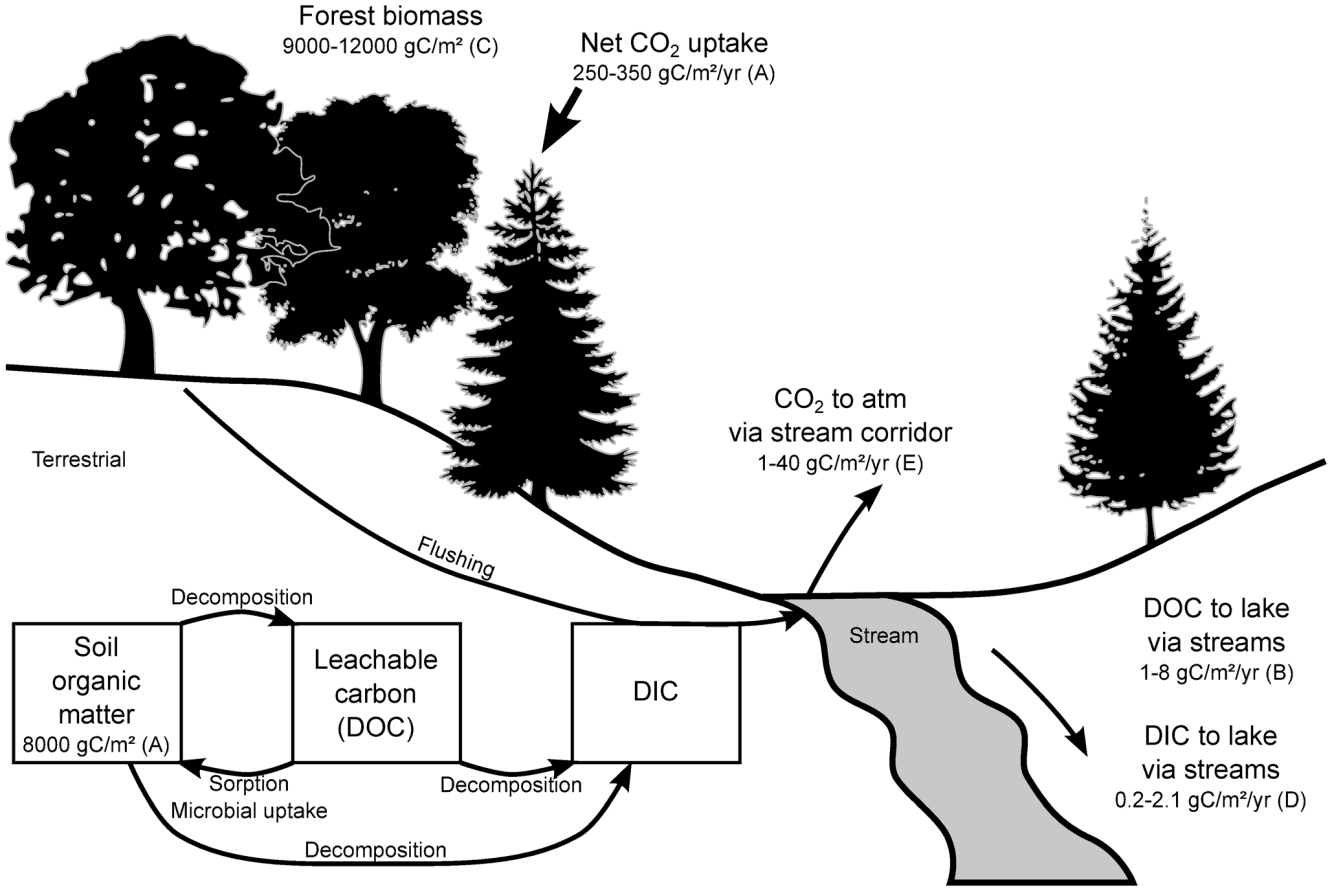
Schematic drawing showing the size of some of the organic carbon pools, carbon fluxes, and important processes affecting DOC, DIC and CO_2_ in terrestrial catchments and aquatic surfaces. Decomposition from soil organic matter includes microbial exudates. Production of DOC, decomposition, microbial uptake/sorption, root respiration, mineral weathering, and flushing are competing processes affecting the export of DOC and DIC via streams in forested catchments. Loss of CO_2_ directly to the atmosphere from the surfaces in the stream corridor can exceed the sum of DIC and DOC export to downstream lakes. Total losses of carbon lost by CO_2_ emissions and dissolved carbon export (DIC+DOC) can be important relative to net CO_2_ uptake by forests from the atmosphere. All rates are per catchment area. (Figure after [42]; A. [62]; B. [38]; C. range for boreal and temperature forests [66]; D. [38], [67]; E. this study.)

One of the study catchments, a small first-order upland stream in a sub-boreal temperate forest (H4-21) [61], lost a median 15 mgC/m^2^/d between its source and mouth (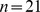, range 3–100 mgC/m^2^/d). This is a flux-weighted loss of 5 gC/m^2^/yr (range 1–40 gC/m^2^/yr) and is comparable to the annual DOC export (1–8 gC/m^2^/yr) from the catchment (Fig. 4). It is also around 10% of catchment net CO_2_ uptake [62]. Unlike DOC, this CO_2_ loss occurred from the small stream surface and was lost directly to the atmosphere before the stream outlet. Furthermore, this CO_2_ loss would not have been quantified at the mouth of the stream using conventional measures of CO_2_ and DIC fluxes [38]. Previous estimates of CO_2_ flux rates from the mouth of Harp Lake streams [21] are much smaller than our estimate. This highlights the fact that measured CO_2_ concentrations decline down the length of streams and CO_2_ loss is underestimated when it is calculated from the sampling points of lowest measured concentrations.

The other study catchment, a typical stream network in Krycklan, Sweden [18], lost upwards of 13–28 mgC/m^2^/d. This estimate is necessarily larger than the CO_2_ concentration based estimated loss of 5.0 gC/m^2^/yr. It is also larger than the DOC+DIC export of 4.6 gC/m^2^/yr and is 14–29% of estimated the net ecosystem exchange of 96 gC/m^2^/yr [18].

This method of using measured in-stream 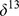
C-DIC values shows that the CO_2_ loss from aquatic surfaces in these boreal and sub-boreal catchments is large and under-estimated. The areal flux rates we present for Harp Lake and Krycklan catchments are similar to those arrived at using much more intensive sampling and different sets of assumptions. This supports the use of a parsimonious model in similar locations. The pattern of measured 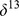
C-DIC values in small streams being much higher than expected shallow groundwater 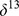
C-DIC values, is present in other datasets [23], [25], [40] and indicates there is a large flux of CO_2_ from headwater streams that has not been included in our continental carbon budgets.

With a measurement at one point in a small stream, the CO_2_ loss can be estimated by this 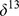
C approach for a certain distance upstream. In H4-21, using a first-order-loss-rate expression (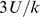, where 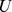 is stream velocity and 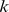 is gas transfer coefficient, [63]), typical gas transfer coefficients (7–25 d^−1^, [21]), typical measured mean discharge (1–20 L/s), typical cross-sectional areas (250–1000 cm^2^), the upstream distance over which the CO_2_ degassing occurred was 375–1375 m.

Grab samples can be used to demonstrate the degree of degassing that has occurred at a given location with a parsimonious model — the simplicity of the DIC–alkalinity–pH–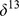
C-DIC calculations means this idea can be easily incorporated into site-specific calculations. Further, stream CO_2_ loss, normalized to catchment area, is likely to be larger than CO_2_ loss from lake surfaces since lake CO_2_ loss is typically less than that half of the DOC input [5], [38], [64].

Headwater stream CO_2_ loss is a reduction in the net ecosystem productivity of boreal and sub-boreal forests. It is a globally important flux since boreal and sub-boreal ecozones are so large [1]. Here, we have shown it can be easily estimated at the catchment scale by combining 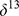
C-DIC measurements with in-stream and groundwater samples. Ultimately, C loss via stream degassing may be required to be integrated into northern hemispheric CO_2_ uptake and loss rates [2], [7], [18], [65].
